# Unveiling cellular and molecular aspects of ascending thoracic aortic aneurysms and dissections

**DOI:** 10.1007/s00395-024-01053-1

**Published:** 2024-05-03

**Authors:** Berta H. Ganizada, Rogier J. A. Veltrop, Asim C. Akbulut, Rory R. Koenen, Ryan Accord, Roberto Lorusso, Jos G. Maessen, Koen Reesink, Elham Bidar, Leon J. Schurgers

**Affiliations:** 1 Department of Cardiothoracic Surgery, Heart and Vascular Centre, Maastricht University Medical Centre, Maastricht, The Netherlands; 2 Department of Biochemistry, Cardiovascular Research Institute Maastricht, Maastricht University, Universiteitssingel 50, 6229 ER Maastricht, The Netherlands; 3 Department of Biomedical Engineering, Heart and Vascular Centre, Maastricht University Medical Centre, Maastricht, The Netherlands; 4 CARIM, Cardiovascular Research Institute Maastricht, 6200 MD Maastricht, The Netherlands; 5 Department of Cardiothoracic Surgery, Center for Congenital Heart Disease, University Medical Center Groningen, Groningen, The Netherlands

**Keywords:** Thoracic aortic aneurysm, Aortic dissection, Extracellular matrix, Vascular smooth muscle cells, Mechanobiology, Biomarkers

## Abstract

Ascending thoracic aortic aneurysm (ATAA) remains a significant medical concern, with its asymptomatic nature posing diagnostic and monitoring challenges, thereby increasing the risk of aortic wall dissection and rupture. Current management of aortic repair relies on an aortic diameter threshold. However, this approach underestimates the complexity of aortic wall disease due to important knowledge gaps in understanding its underlying pathologic mechanisms.

Since traditional risk factors cannot explain the initiation and progression of ATAA leading to dissection, local vascular factors such as extracellular matrix (ECM) and vascular smooth muscle cells (VSMCs) might harbor targets for early diagnosis and intervention. Derived from diverse embryonic lineages, VSMCs exhibit varied responses to genetic abnormalities that regulate their contractility. The transition of VSMCs into different phenotypes is an adaptive response to stress stimuli such as hemodynamic changes resulting from cardiovascular disease, aging, lifestyle, and genetic predisposition. Upon longer exposure to stress stimuli, VSMC phenotypic switching can instigate pathologic remodeling that contributes to the pathogenesis of ATAA.

This review aims to illuminate the current understanding of cellular and molecular characteristics associated with ATAA and dissection, emphasizing the need for a more nuanced comprehension of the impaired ECM–VSMC network.

## Introduction

**Fig. 1:**
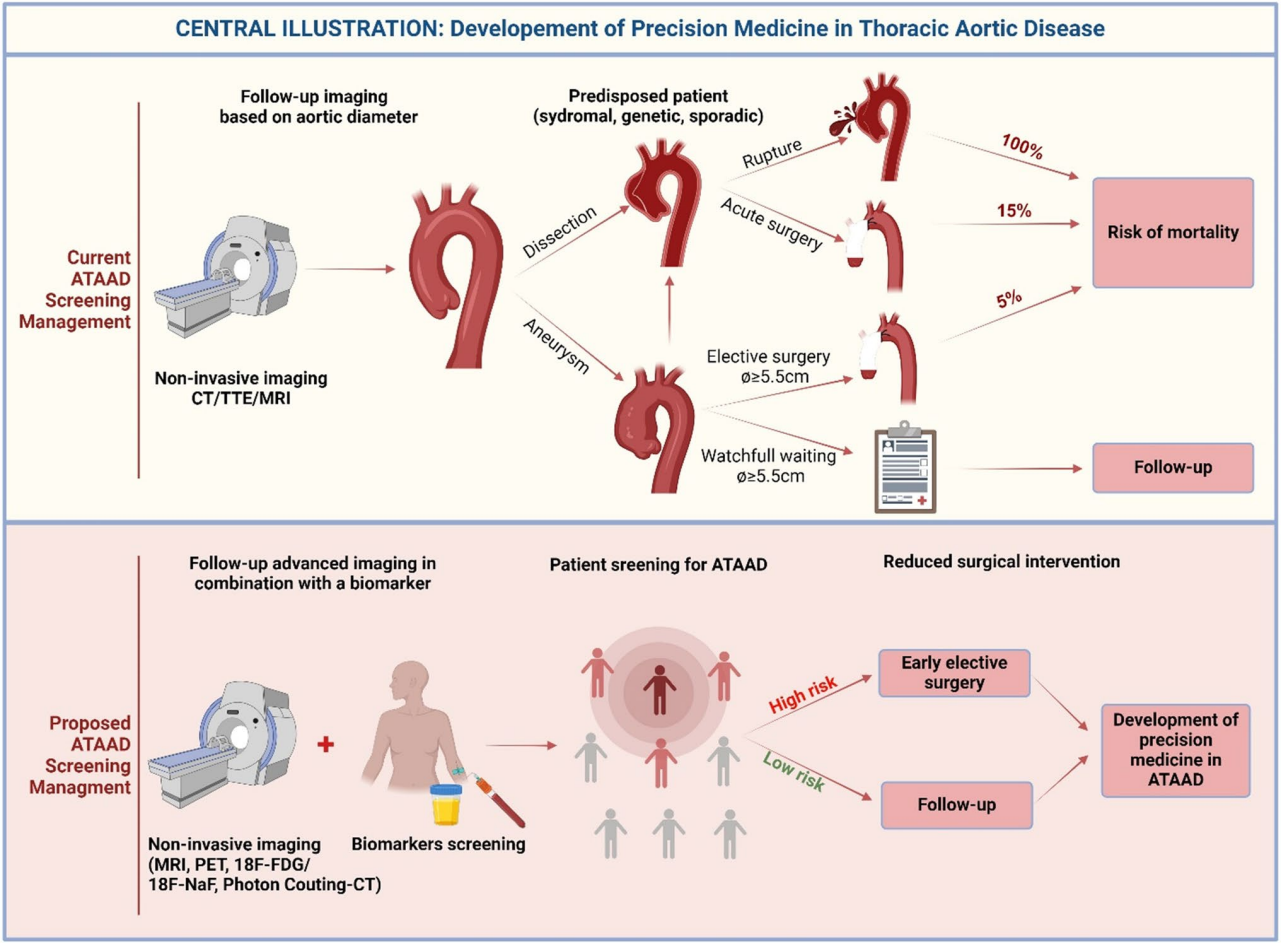
**Central illustration:** Development of Precision Medicine in Thoracic Aortic Diseases gives a summary of the current clinical patient screening management based on diameter threshold (including the risk of aneurysm and rupture) and the proposed screening management with the use of clinical biomarkers as an add-in to imaging modalities to prevent invasive surgical repair and high-risk of mortality

**Fig. 2:**
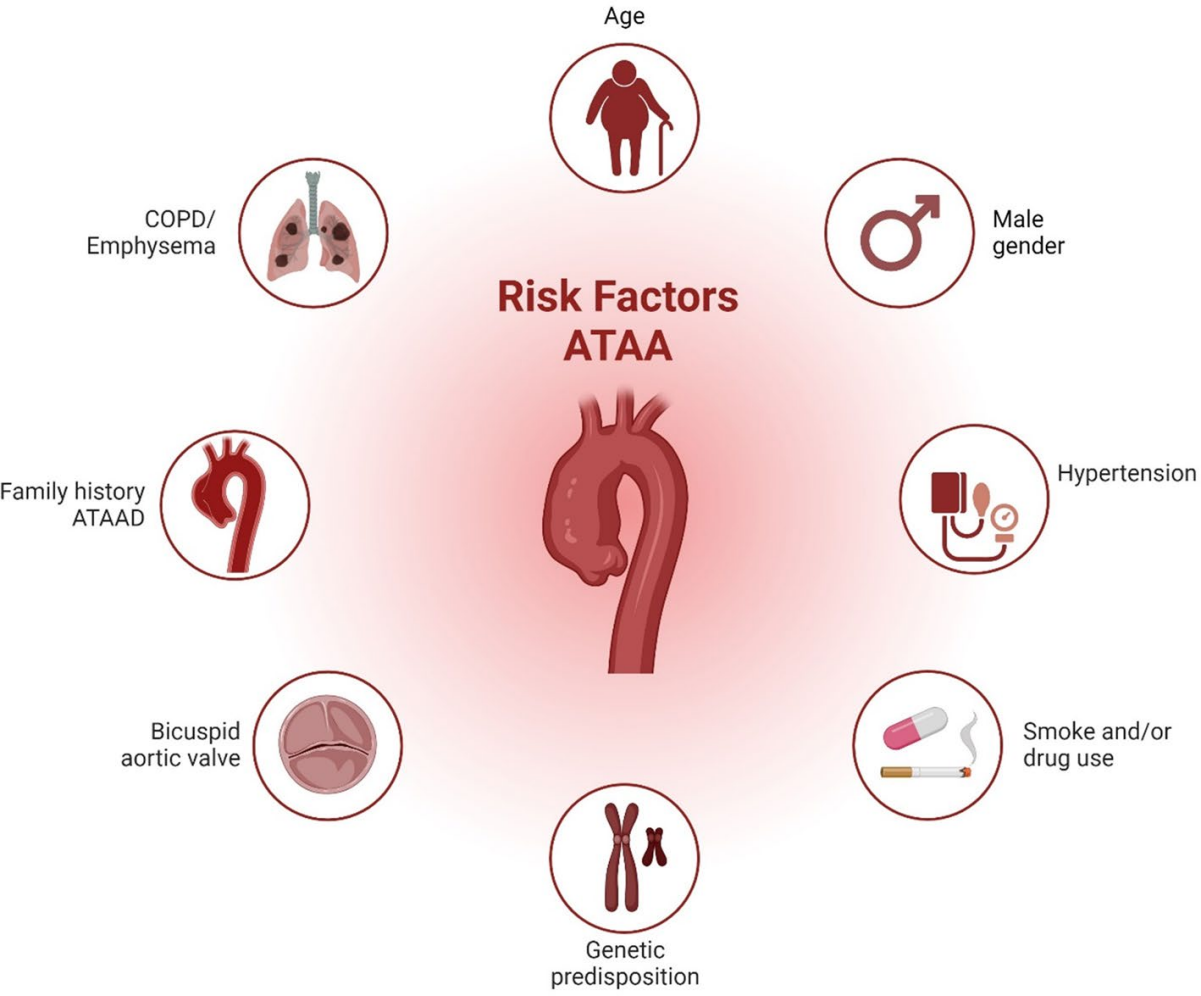
Schematic overview of major clinical risk factors in ATAA. Besides genetic syndromes, connective tissue disease, and bicuspid aortic valve morphology, these include hypertension, smoking, male sex, age, and COPD

An ascending thoracic aortic aneurysm (ATAA) is a localized dilation in the proximal segment of the aorta. Aortic aneurysms represent weakened areas within the aorta, posing a significant risk of tearing or rupturing and resulting in severe, potentially life-threatening internal bleeding. If left untreated, ATAA can lead to severe complications such as aortic dissection (ATAAD) and rupture, with mortality rates of 50% within 24 h, including 21% mortality in patients who arrived alive in the hospital (Fig. 1) [137]. Known risk factors for ATAA development include advanced age > 65 years, systemic hypertension, and male sex (Fig. 2) [40]. Indeed, ATAA is diagnosed more frequently in men, which also is reflected in 70% of individuals with ATAAD [152]. On the contrary, ATAA severity has been indicated to be worse in women compared to men with faster aneurysm growth [32] and increased in-hospital mortality rates [134]. The estimated incidence of ATAA ranges from 5–10 per 100,000 individuals/year [37, 156], with a currently increasing trend [107]. Compared to abdominal aortic aneurysm (AAA), ATAA exhibits different flow patterns [176], regional variations [185], and developmental origins [193].

Notably, prophylactic treatment options of ATAA are limited to aortic surgery in which a diameter threshold of 5.5 cm and aortic diameter growth rate ≥ 0.3 cm/year are recognized as cut-off values [37]. However, it is recognized that most patients develop ATAAD before reaching these thresholds (Fig. 1) [75, 161, 163].

Therefore, clinical biomarkers, representing early detection of patients at risk of ATAAD are highly anticipated. From an etiological point of view, less than 30% of all ATAA cases are genetically triggered, and thus more than 70% are sporadic or degenerative [35]. It is tempting to speculate that unknown (epi)genetic factors play a key role in initiating and progressing degenerative ATAA.

In patients with bicuspid aortic valves (BAV) or genetic mutations, ATAA is more commonly observed at a younger age [198]. Heritable ATAA has been associated with over 15 genes, including those encoding ECM-proteins such as fibrillin 1 (*FBN1*), type III collagen (*COL3A1*), or transforming growth factor-β (TGF-β) receptor proteins such as *TGFBR1* and *TGFBR2* [204]. It also involves VSMC proteins such as smooth muscle cell actin (*ACTA2*), myosin heavy chain 11 (*MYH11*), myosin light chain kinases (*MYLK*), and protein kinase cGMP-dependent type 1 (*PRKG1*) [159].

Furthermore, arteries are exposed to wall shear stress (WSS), which is induced by blood flow and exerted at the valvular and vascular endothelial layers [10]. Unequal distribution of WSS at the outer curvature of the ascending aorta has been associated with degenerative ATAA [171]. Blood pressure also exerts a key hemodynamic influence, i.e., wall stress, on the integrity of aortic wall tissue [61].

In the medial layer, vascular smooth muscle cells (VSMC) play an important role in vascular remodeling, exhibiting characteristic plasticity to adapt to changing flow and pressure conditions, e.g., by switching between a contractile and a synthetic phenotype [177]. VSMCs are further supported by the extracellular matrix (ECM) which plays a crucial role in regulating mechanical behavior and resilience, providing elasticity, and imparting arterial wall strength [2]. Once the ECM–VSMC network is disrupted, mechanosensing and mechano-signaling are impaired, leading to VSMC phenotype switching [153]. A compromised structure and function of the ECM leads to mechanical abnormalities and functional changes at the tissue level associated with aortic disease [208]. Progressive loss of arterial wall strength eventually culminates in the development of ATAA and even ATAAD.

There is an urgent need to identify novel biomarkers to screen patients at high-risk for ATAAD. Here, we summarize the current literature on the pathophysiology of ATAA and ATAAD with a focus on ECM–VSMC dysfunction. We highlight gaps in current diagnostic approaches, as well as recommend potential clinical biomarkers that may contribute to advancing our understanding of the development of early-stage ATAA, ultimately to predict and prevent morbidity and mortality associated with ATAAD.

## Challenges in clinical management

In clinical practice, the characterization of ATAA is predominantly confined to diameter measurements. The aortic diameter is the principal decision-making criterion for surgical intervention within the Multidisciplinary Aortic Team. According to the latest clinical guidelines (EACTS/STS) for aortic diseases published in 2024, surgical replacement of the aorta is recommended when the aortic diameter is greater than 5.5 cm [37]. In high-risk patients with the presence of connective tissue disorders (e.g., Marfan or Loeys-Dietz), earlier intervention is recommended (≥ 4.5 cm). Patients with low-risk are monitored by imaging every year for timely detection of the surgical threshold (Fig. 1). However, up to 96% of ATAAD occurs in vessels with diameters below the surgical interventional threshold (< 5.5 cm) and 60% of ATAAD occurs in aortas with normal diameters (< 4 cm) [180]. Also, most patients with ascending aortic aneurysms of > 4 cm show little to no further growth during annual follow-up [1]. This is a major unmet clinical challenge for cardiologists and cardiac surgeons in assessing and managing ATAA.

Recently, several new parameters such as aortic elongation and aortic volume have been identified as potentially important predictors of ATAAD [74, 87]. Although these parameters alongside other morphologic characteristics of the vasculature such as the vertebral artery tortuosity show promising results [147], they still represent patients at a later stage of disease development and earlier detection of patients at risk requires a holistic approach implementing multi-scale analysis including vessel wall characterization.

In recent years, imaging modalities such as computed tomography (CT) combined with positron emission tomography (PET), using fluorodeoxyglucose (18F-FDG) or 18F-sodium fluoride (18F-NaF) administration provide geometrical, molecular, and functional information of aortic disease [56]. Several studies showed promising results regarding aortitis [80, 81], aneurysm growth and future clinical events [103], atherosclerosis [179], and detecting malignant tumors of the aorta [199]. Photon Counting-CT is another promising technique, which is still in clinical trial, yet has proven its clinical value regarding improved spatial resolution, and optimized spectral imaging [221]. Furthermore, this method offers precise tissue characterization and improved perfusion imaging while minimizing radiation exposure.

Blood-flow characteristics play a key role in ATAA formation, with effects on endothelial cell (EC) homeostasis and the response of VSMCs. Therefore, a functional assessment of detailed hemodynamic measurements is required to investigate flow characteristics and biomechanical forces. Four-dimensional flow magnetic resonance imaging (4D)-flow MRI, where phase-contrast methods are used to encode blood flow velocities along all dimensions in the aorta [42, 110], has been introduced as a powerful non-invasive technique in cardiovascular imaging for the assessment of local WSS [30]. WSS, which refers to the force per unit area exerted by moving fluid in the vessel, can be estimated as a product of wall shear rate (WSR) and local blood viscosity. Yet, it has not been validated as a clinical screening tool. One of the limitations of 4D flow MRI is insufficient spatial resolution which may underestimate WSS values and affect the accuracy of flow patterns [86]. Broader application of 4D flow MRI has been impeded by long scan times, costs, and data processing and analysis requiring special software. These obstacles hinder both reproducibility and clinical application [131]. Nevertheless, some longitudinal studies showed that 4D flow MRI can be used as a predictive tool to distinguish low and high WSS in ATAA patients with BAV [14, 20, 67, 144] or without BAV [171] compared to healthy volunteers.

Recently, the left ventricular outflow tract angle (LVOT-angle) has been a region of interest in ATAA pathology. Aortas less aligned to the axis of the heart were associated with ATAA [97]. Another study showed a positive correlation between LVOT-aortic angle and WSS on the outer curvature, indicating that increased LVOT-aortic angles (> 58.5º) were linked to elevated levels of WSS [184]. Geometrical biomarkers combined with flow patterns may improve the prediction of ATAA at risk or those who need an earlier intervention.

## Pathophysiology

### VSMC phenotypic switching in ATAA

In the adult arterial vessel wall, VSMCs are present in both the contractile and synthetic phenotype. Contractile VSMCs are connected via integrins to the ECM and are in a quiescent, non-proliferative state and facilitate contraction and dilation of resistance vessels and microvasculature, thereby regulating blood flow [160]. Contractile phenotype markers include smooth muscle-myosin heavy chain 11 (MYH11), calponin, smooth muscle-22 α (SM22α), and α‐smooth muscle actin (ACTA2) (Table 1). After VSMCs are released from the ECM, integrins trigger intracellular signaling and regulate VSMC phenotypic switching from “contractile” to “synthetic” phenotype. Synthetic VSMCs are characterized by reduced expression of contractile markers (Table 1) [57], and increased production of matrix metalloproteinase (MMP) thereby shifting the balance towards cell migration and ECM remodeling [27]. This transition of VSMCs toward synthetic phenotype can be assessed by integrin detection by flow cytometry, immunocytochemistry, and immunoprecipitation [88, 194].

**Table 1:** Characteristics of widely used protein markers for VSMCs, distinguishing between contractile and synthetic phenotypes

Protein marker	Gene code	Subcellular localization	Function	VSMC Phenotype specificity		Expression in ATAAExpression in ATAA	Ref
				Contractile	Synthetic	(± /mut)	
α-smooth muscle actin	*ACTA2*	Contractile filaments	Cellular contraction	+	-	−/mut	Guo et al., 2007 Gillis et al., 2013 Branchetti et al., 2013
Smooth muscle-myosin heavy chain	*MYH11*	Contractile filaments	Cellular contraction	+	-	−/mut	Zhu et al., [235] Gillis et al., 2013
Smooth Muscle 22α	*SM22*α	Actin-associated	Cellular contraction	+	-	-	Ignatieva et al., 2017
SM-calponin	*CNN*	Actin-associated/ cytoskeleton	Cellular contraction/ signal transduction	+	-	-	Grewal et al., 2014
Smoothelin (B)	*SMTN*	Actin-associated	Cellular contraction	+	-	-	Grewal et al., 2014 Branchetti et al., 2013
h-Calmodulin	*CALM*	Cytoplasm/nucleus	Cellular contraction	+	-	−/mut	Wang, et al. 2010
h-caldesmon	*h-CD*	Actin-associated	Cellular contraction	-	+	NA	
Vimentin	*VIM*	Actin-associated/ cytoskeleton	Cellular contraction	-	+	+	Branchetti et al., 2013
S100 calcium-binding protein A4	*S100A4*	Cytoplasm/nucleus	Cellular proliferation	-	+	+	Cao et al., 2013
Osteopontin	*OPN*	Nucleus	Cellular proliferation	-	+	+	An et al., 2017

*(h) High molecular weight; (* +*) present;( -) reduced;( -/mut) reduced or mutation; NA no conclusive evidence*

**Fig. 3:**
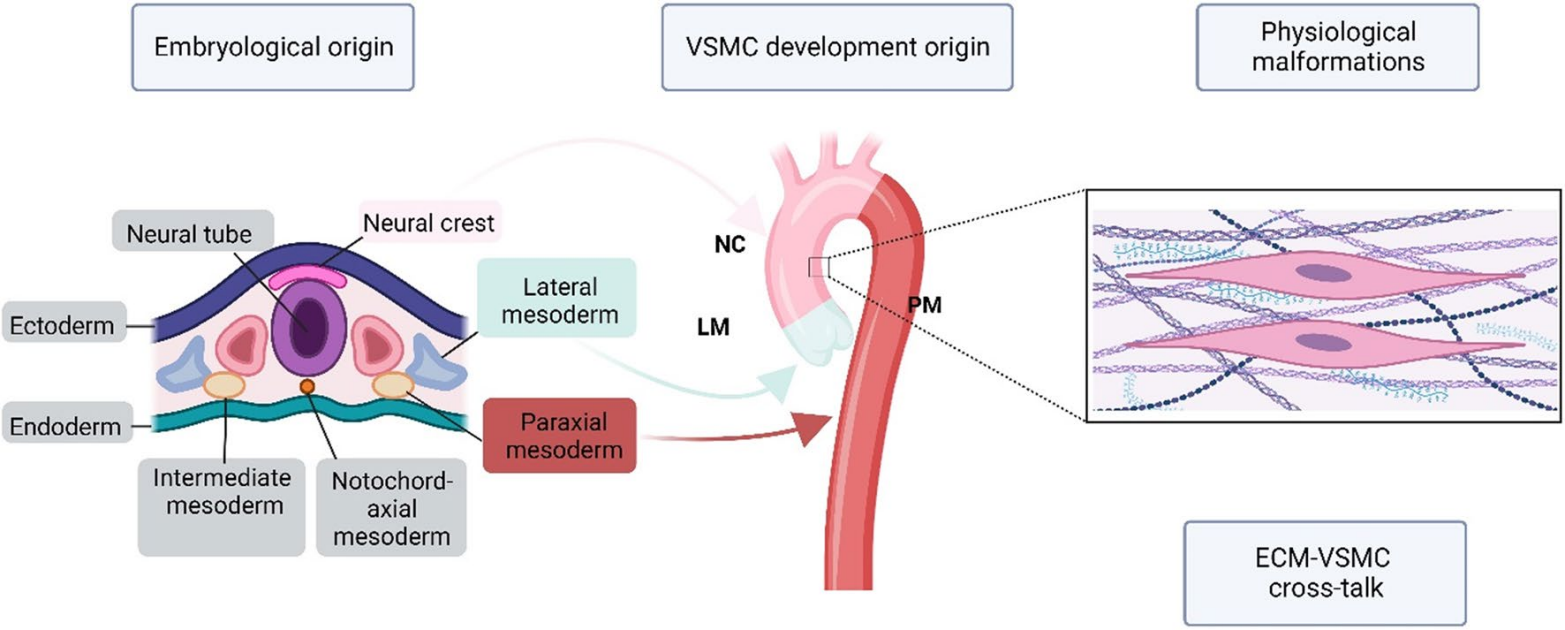
Regional heterogeneity and embryological diversity within human aorta: *LM* lateral mesoderm (*green*, located in aortic root), *NC* neural crest (*pink*, located in ascending/arch), *PM* paraxial mesoderm (*red*, located in descending aorta)

In larger and so-deemed ‘elastic’ arteries and especially in the aorta, the precise role and relevance of the contractile phenotype and contractile responses to mechanical vessel wall stress and biomechanical stretch are not fully understood. Notably varying sites of the aorta are derived developmentally from different embryonic origins. VSMCs of the aortic root are predominantly derived from the lateral mesoderm. In contrast, VSMCs of the aortic arch are derived from the neural crest, and the descending aorta VSMCs originate from the paraxial mesoderm (Fig. 3) [193]. Notwithstanding there is overlap in the descending aorta wherein spatially distinct domains, have been noted by lineage fate tracing in mice [186]. However, the use of lineage-specific differentiation to VSMCs from human-induced pluripotent stem cells (hiPSCs) has yielded distinct cellular phenotypes suggesting lateral mesoderm malformations correlating to root dilation in Loeys-Dietz and neural crest VSMCs in Marfan associated ATAA [65, 234].

Disruption of homeostasis for vascular repair may result in a key role of VSMCs contributing to vascular pathology. Owens and colleagues showed that the high plasticity of VSMCs facilitates phenotypic switching towards synthetic VSMCs to adapt to environmental stress [160]. Furthermore, it has been shown in a co-culture model of ECs-VSMCs, that altered flow on ECs already induces a synthetic VSMC phenotype [183]. Also, the local inflammatory cascade can induce a phenotypic switch of VSMCs and transform them into synthetic VSMCs with fibroblast-like characteristics [195]. This phenotypic transition often leads to an increase in MMP production [192].

### The impact of aberrant wall shear stress on mechanotransduction

Mechanobiology implies that cellular mechanosensing and ECM regulation are critical for maintaining mechanical homeostasis and proper vascular function [80]. Mechanotransduction is the biochemical process through which ECs and VSMCs convert mechanical stimuli through the cytoskeleton, leading to intracellular responses and extracellular changes [166]. In addition, EC integrins play a crucial role in the mechanotransduction of VSMC contractility [151].

Shear stress activates EC integrins by switching them to an active conformation, thereby increasing affinity to ECM proteins [220]. During systole, ECs, and VSMCs experience both longitudinal and circumferential mechanical deformation (‘strain’). The disruption of elastin-VSMC connections plays a critical role in aneurysm formation, not only by damaging the structural integrity of the aortic wall but also by altering cellular processes such as mechanotransduction and cytoskeletal remodeling of VSMCs [241]. ECs regulate these processes by activating mechanosensors, including vascular endothelial growth factor receptor 2 (VEGFR2), vascular endothelial-cadherin (VE-cadherin), and platelet EC adhesion molecule (PECAM-1) [232]. It has been demonstrated that ECs derived from the aortic wall of an aneurysm present decreased levels of VE-cadherin, von Willebrand factor (vWF), and PECAM-1 [134]. These decreased levels disrupt mechanotransduction and induce macrophage infiltration in the media and adventitia through nuclear factor-kB NF-κB activation [190, 200].

Different studies on BAV patients with ATAA have confirmed increased WSS in the greater curvature of the ascending aorta [45, 46]. In this greater curvature region, there was evidence of increased medial degeneration with reduced collagen type I and III and increased VSMC apoptosis [45, 46]. Moreover, it has been demonstrated that the WSS effect on media degeneration and VSMC phenotype change (expressing synthetic marker MYH10) has been shown in ATAA patient samples [102]. High WSS is a frictional force at the EC surface produced by blood flow which induces impaired mechanotransduction leading to vascular remodeling and potentially ATAA formation [192]. The initial sensing event and transduction of the mechano-signaling pathway are as follows: under constant laminar flow, the mechanosome is quiescent and inactive. However, when shear stress changes, the mechanosome consisting of PECAM-1, VEGFR2, and VE-cadherin triggers the activation of NOX2 and eNOS, resulting in the release of ROS and NO [31]. In addition, increased WSS corresponded with changes in the ascending aorta using pre-operative WSS mapping [71]. Here, increased elastin degradation in regions of high WSS, as well as increased TGF-β1 and MMP-1, MMP-2, and MMP-3 were reported [71].

However, the relation between WSS and gene expression, and protein synthesis remains unclear and needs further investigation. Such information is key for fundamental research on shear stress–mechanotransduction mechanisms. Clinically, it could aid in explaining why certain patients with an aortic diameter below the current intervention criteria still develop acute aortic complications.

### ECM degradation

The major histopathological features associated with ATAA are abnormalities of cellular and matrix constituents of the media. These include elastin degradation and fragmentation, collagen disorganization, and loss of VSMC number [132]. In addition, mucoid ECM accumulation is a common pathologic finding in TAA and can serve as a marker for ECM degradation [72]. Furthermore, elastic fiber fragmentation has been reported to be greatest in the proximal aneurysmal ascending aorta compared to the middle or distal regions [197]. Furthermore, fibulin-4 (Fbln4), a component of elastic fibers essential for maintaining aortic wall integrity has been implicated in aneurysm formation. Loss of Fbln4 was associated with significantly upregulated levels of thrombospondin-1 (Thbs1), a homotrimeric glycoprotein [227]. Fhbs4 expression is induced by mechanical stretch resulting in disruption of elastin-VSMC connections and decreased mechanosensing. Under physiologic conditions, VSMCs maintain ECM homeostasis by a balanced secretion of MMPs and their inhibitors TIMPs to maintain a load-bearing mechanical state.

#### The disturbed balance between TIMPs/MMPs

In ATAA, dysfunction of VSMCs causes an imbalance between the production of active MMPs [219], especially MMP-2 and MMP-9 [83, 123], and a decreased expression of TIMPs, mainly TIMP-1[6]. Other proteolytic enzymes have also been found to modulate both ECM and VSMC function in ATAA, such as A-disintegrin metalloproteinase (ADAMTS-1, ADAMTS-4) [172]. Recent data have demonstrated that MMP-1, -2, -3, -9, -12, and -13 play roles in the progression of ATAA [111, 114, 169, 216]. Specifically, MMP-2 and MMP-9 are known to degrade collagen fragments and MMP-2, MMP-9, and MMP-12 elastin fragments [122]. This, in turn, facilitates the disengagement of VSMCs resulting in aortic tissue remodeling [4, 139, 177, 181]. In a mouse model deficient in MMP-2, ANG-II infusion resulted in exacerbated ATAA. The same study unveils the dual role of MMP-2 in both degrading and synthesizing ECM, showcasing its multifaceted role in tissue remodeling [191]. Nevertheless, MMPs and TIMPs are widely distributed throughout physiologic processes in different organs, suggesting that MMP and TIMP blood levels may not represent reliable biomarkers to correlate with aortic aneurysm levels.

In individuals with BAVs, MMP-2 levels are higher compared to those with TAVs. However, MMP-13 levels in TAV samples are significantly higher than in BAVs [84]. MMP-13 is a member of the collagenase subgroup within the MMP family, and previous studies have shown its upregulation in both human AAA [130, 206] and ATAAD [232] tissues. MMP-13 is primarily synthesized by VSMCs present in the aortic wall [130] triggered by JNK, ERK, and p38 kinases of the MAPK family. It not only degrades elements of the aortic collagen network, such as type I and III collagen [34], but also proteins within the elastic fiber networks, such as fibrillin 1[9], fibronectin [196], and decorin. This degradation has a significant impact on the structure of the ECM, potentially contributing to the growth and dissection of the aorta. A separate study provided initial evidence indicating that the collagenase MMP-13 contributes to aneurysm development in mouse models of Marfan syndrome. Pharmaceutical inhibition of MMP-13 in Fbn1 GT8 Marfan mice effectively prevents aortic root dilatation, underling the relevance of MMP-13 as a potential therapeutic target for managing aortic aneurysms [237].

In ATAA, a decrease in the elastin-to-collagen (ELN/COL) ratio is associated with increased aortic stiffness [108]. Typically, the ELN/COL ratio in the media of healthy aorta is some 1.7–1.9, whereas in the media of ATAA aortas, ELN/COL ratios are as low as 0.83–0.81 [223]. Increased collagen expression in the vasculature is most likely a compensatory response to elastin degradation and thus vascular remodeling [19] which results in the thickening of the arterial wall and increased vascular stiffness [78, 236].

### Vascular calcification in ATAA

Vascular calcification has been suggested as a potential measure strongly associated with atherosclerosis [190], ATAA, and AAA [13]. It has been reported that ATAA patients develop extensive medial calcification associated with a phenotypic switch of VSMCs into osteoblastic-like- cells therefore creating a pro-calcifying environment [151, 224]. There is emerging evidence indicating that Krüppel-like factor 4 (KLF4), a potent tumor repressor, regulates the transition of VSMCs into osteogenic phenotypes in both murine and humans [5]. Initial calcifications, often referred to as micro-calcifications, typically measure less than 50 μm in size [98] and primarily originate from extracellular vesicles of osteochondrogenic VSMCs [89]. Increased medial micro-calcification was associated with mild and moderate histopathological degeneration (mild/moderate elastin fragmentation) [53]. Instead, patients with severe histopathological degeneration (severe elastin fragmentation), exhibited reduced medial micro-calcification [53]. This mechanism relies on intact elastin fibers for the deposition of micro-calcification.

In the early stage of the disease, micro-calcification can be measured ex vivo by the expression of osteogenic VSMC markers alkaline phosphatase (AP) and osteopontin (OP), or in vivo using 18F-sodium fluoride autoradiography (18F-NaF) [53]. The deposition of micro-calcification in combination with the local fragmentation of elastin fibers is associated with an increased risk of aortic wall rupture, as a result of stiffened regions of the soft ECM in the vessel wall [53, 220].

Matrix Gla-protein (MGP) is an inhibitor of medial micro-calcification that is widely recognized for its importance. It is predominantly secreted by VSMCs and is a vitamin K-dependent protein (VKDP). For MGP to become biologically active, it must undergo post-translational modification via vitamin K-dependent carboxylation by the enzyme gamma-glutamyl carboxylase (GGCX) [21]. Oral anticoagulation or a deficiency in vitamin K can result in inactive MGP. This is indicated by increased levels of dephosphorylated undercarboxylated MGP (dp-ucMGP) in the circulation. It should be noted that dp-ucMGP is a biomarker of vitamin K status and has been related to vascular calcification [91]. In humans, MGP deficiency is known as Keutel Syndrome, a genetic condition characterized by soft tissue calcification [79]. It has been demonstrated that MGP deficiency in humans may exhibit a gradual onset of calcification in both arteries [91] and heart valves [29]. Further, there appears to be a correlation between MGP deficiency and elastin calcification [188]. This data indicates a link between impaired carboxylation of MGP and the development of calcification starting around elastin fibers in the tunica media of patients who underwent percutaneous coronary intervention. It also implies the crucial role of vitamin K in activating MGP to effectively prevent vascular calcification.

## Regulatory pathways

### Arterial remodeling modulated by TGF-β signaling

Various molecular pathways involved in the synthesis of the ECM exhibit alterations in ATAA. This is evident for example through the upregulation of fibrogenic growth factors like TGF-β, platelet-derived growth factors (PDGF), and connective tissue growth factor (CTGF) [77, 95, 174]. Platelet-derived growth factor-BB (PDGF-BB) and TGF-β serve as pivotal mediators in VSMC phenotypic switching [27, 214, 235]. For example, it has been reported that in Marfan syndrome, an increased expression of TGF-β in VSMCs, results in the activation of SMAD3 and Erk signaling contributing to aneurysm progression [149, 229]. Other factors that alter the aortic wall integrity include angiogenic factors including angiopoietin-1, angiopoietin-2, thrombospondin-1, and fibroblast growth factor-1 [100].

Activation of TGF-β can be triggered by multiple factors, such as thrombospondin [187], and reactive oxygen species (ROS) [51, 120]. Increased expression of TGF-β in VSMCs of patients with Marfan syndrome has been associated with increases in ROS production [93, 229]. In addition, TGF-β activation occurs through the proteolytic degradation of the latent TGF-β complex by MMP-2 and MMP-9. Also, integrin αV can activate TGF-β1 by establishing a close connection between the latent TGF-β complex and MMPs [222, 230]. It thus appears that TGF-β1 induces VSMC senescence via ROS-mediated activation of NF-κB signaling, potentially contributing to aneurysm formation in Marfan patients.

Another pathway involved in TGF-β signaling is the PRKG1 which regulates VSMC relaxation through type I cGMP-dependent protein kinase (PKG-1) [69]. In ATAA, the impaired PRKG1 pathway inhibits Rho-associated protein kinase (ROCK), ensuring the myosin light chain remains in a relaxed state and leading to a reduction in VSMC contractility [202].

Moreover, SMAD3 is a critical transcription factor in the TGF-β signaling pathway, regulating VSMC differentiation and matrix deposition. Heterozygous SMAD3 mutations increase the risk of aortic root aneurysms, which may progress to type A aortic dissections without surgical intervention [62]. Hence, TGF-β through SMAD3 signaling stimulates the proliferation and differentiation of neural crest-derived VSMCs in the ascending aorta [207]. Patients with Loeys–Dietz syndrome (LDS) are characterized by mutations in genes encoding for TGF-β receptors 1 and 2 [214]. In patients with LDS, increased secretion of TGF-β ligands activates TGFBR1/TGFBR2 complexes and enhances TGF-β signaling [60]. In vitro, VSMC explants from patients with heterozygous mutations in TGFBR2 showed decreased expression of VSMC contractile proteins and displayed a synthetic VSMC phenotype [85]. Further, in human ATAAD genetic variants in SMAD4, a secondary messenger of the TGF-β pathway, correlate with VSMC apoptosis, reduced contractile markers, and ECM degradation [49].

### Down-regulation of YAP in response to mechanical stress

Involvement of the Hippo pathway in ATAA has been seldom reported, which is surprising due to the pivotal role of mechanobiological processes in aneurysm formation. The Hippo/Yes Associated Protein (YAP) signaling pathway is a highly evolutionary conserved mechanism with a central role in regeneration, proliferation, migration, and cell fate biology [59]. By initiating a cascade of several kinases, YAP, and its WW-domain-containing transcription regulator 1 (WWTR1; also known as and hereafter referred to as TAZ) are controlled in mammalian cells. Diverse upstream biomechanical and mechanobiological cues, such as WSS, vascular stiffness, or hypertrophic responses, regulate the Hippo/YAP signaling pathway which results in a dynamic interaction between vascular cells and their surrounding ECM [73]. YAP was identified as a key transcription factor in a mouse model, driving a pivotal adaptive response mechanism that appeared to be critical for maintaining aortic homeostasis and preventing ATAAD in mice [231]. This study demonstrates that YAP signaling plays a crucial role in the vascular remodeling of aneurysmal specimens, as evidenced by the elevated medial thickness, indicating an adaptive response to increased wall stress.

Mechanical stress-induced YAP down-regulation has been reported in human aortic samples from patients with type A aortic dissection [116]. The induced aortic stress initiated a YAP nuclear translocation which led to the protection of the aorta from medial degeneration and the development of aneurysm and dissection.

The Hippo/YAP pathway alters ECM production or degradation and the growth, death, and migration of VSMC and endothelial cells, which contributes to vascular remodeling in aortic aneurysms. A similar phenomenon was observed in a mouse BAPN-induced Stanford type A aortic dissection model [92].

Via KEGG pathway identification, a series of different target genes and pathways were identified in human tissues linked to aneurysm formation, including the Hippo pathway [3, 23]. In line with the notion that Hippo’s transcriptional activator YAP and extracellular signal-regulated kinase 1/2 (Erk1/2) activities are related, Bertrand et al. showed that impaired mechanotransduction results in a hyperinduction of mechanical stress, subsequently activating YAP and increasing Erk1/2 signaling [17].

In addition, the Hippo pathway is a convergence point of cellular signaling with multiple major pathways, including Wnt/β-catenin, insulin-like growth factor (IGF), Phosphoinositide 3-kinases—RACα serine/threonine-protein kinase (Pi3K-AKT), and mammalian target of rapamycin (mTOR) signaling [138]. The regulation of YAP through these diverse pathways may expand the known mechanisms of vascular remodeling regulated by the Hippo/YAP pathway.

### Alteration in notch signaling

**Fig. 4:**
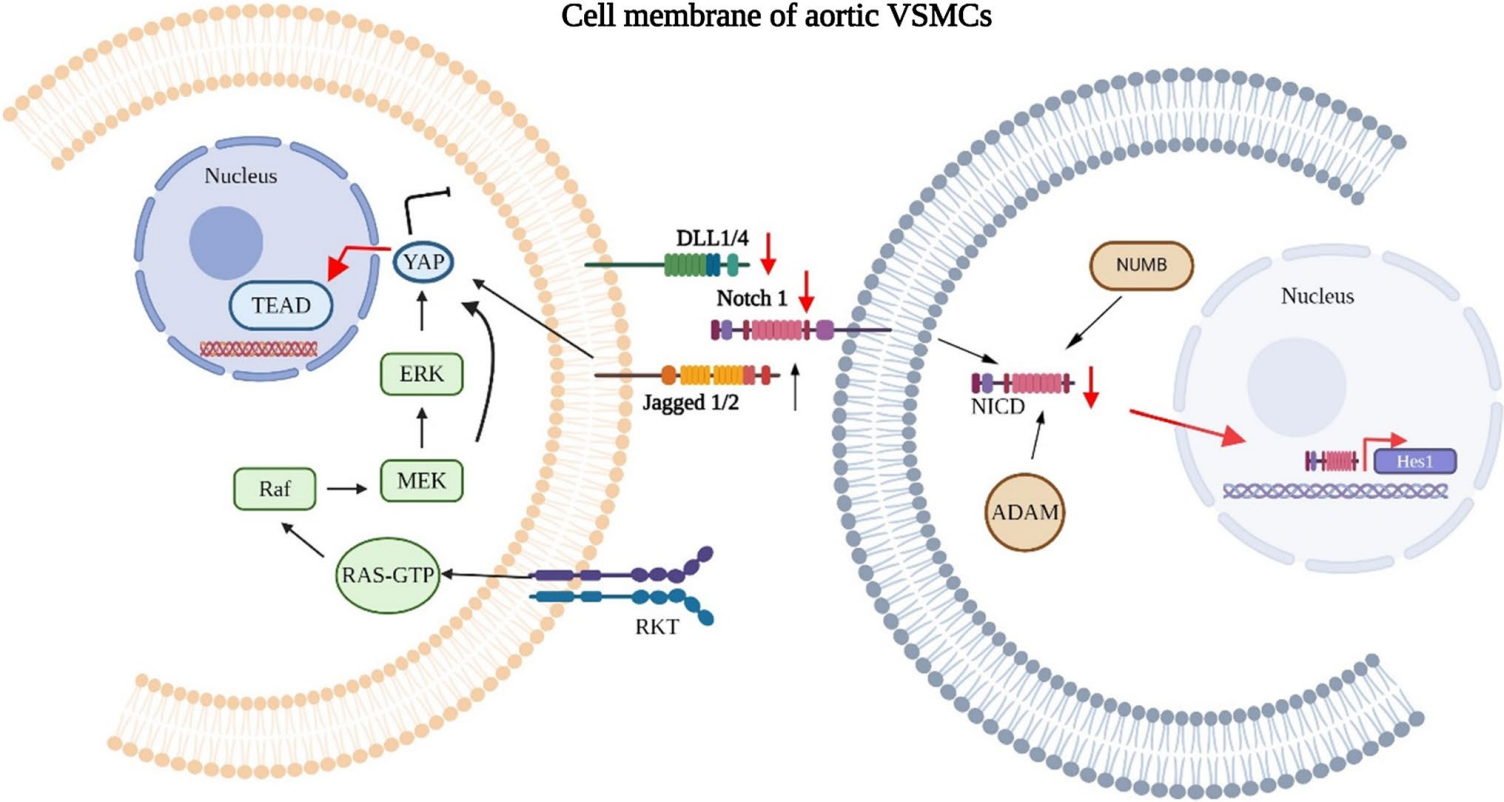
An overview of suggested intra- and intercellular mechanosensitive pathways involved in vascular homeostasis (*black arrows*) and pathologic condition in ATAA (*red arrows*). In impaired vascular homeostasis, intracellular pathways leading to nuclear translocation of Hippo pathway effector YAP inducing proliferation. Downregulation of Notch1 and DLL1/4 proteins can have significant effects on cellular function and may impact various physiologic processes. When Notch1 and DLL1/4 proteins are downregulated, it can lead to reduced activation of Notch Intracellular Domain (NICD). In this scenario, the transcriptional repressor Hes1 fails to activate, which is pivotal for VSMC proliferation. Therefore, dysregulation of the Notch signaling pathway could potentially contribute to pathologic processes involved in the development and progression of ATAA

The notch signaling pathway in ATAA is not well-defined in patients. However, several in vitro investigations have illustrated the involvement of Jagged–Notch signaling in impaired mechanosensing, resulting in the initiation of phenotype switching and differentiation of VSMCs [121, 148]. This compromised Notch signaling has been observed in human tissues originating from both ATAA and ATAAD [128, 238]. The Notch pathway and Wnt signaling are involved in vascular development and physiology and play a critical role in controlling phenotypic switching of VSMCs [11, 48, 119]. Through interactions with TGF-β [222] and PDGF [94], Notch signaling regulates the migration and differentiation of VSMCs. In addition, Wnt inhibitory factor-1 acts as an inhibitor, suppressing PDGF-BB-induced proliferation of VSMCs [209]. These pathways collectively contribute to the intricate regulation of VSMC behavior. Key mediators in this highly conserved pathway in ATAA are Notch 1, Notch 3, and Jagged 1 (Fig. 4). Notch 1 and Notch 3 [64, 127] regulate the migration and proliferation of VSMCs in vascular injury models, and mutations in these receptors lead to defects in VSMC development [24, 118, 218].

There is supporting evidence indicating that the mechanosensor Piezo1 plays a crucial role in responding to shear stress [28]. This response involves the activation of a disintegrin and metalloproteinase domain-containing protein 10 (ADAM10), ultimately leading to the cleavage of Notch1, which then translocates to the nucleus, initiating the transcriptional activation of downstream targets [28]. These cellular processes are crucial in maintaining vascular integrity and may be implicated in the context of ATAA where structural changes occur.

Importantly, Notch is severely affected by biomechanical stimulation, inhibiting VSMC proliferation, while increasing apoptosis [148]. Although Notch is linked to vascular development, there is still no evidence of the precise mechanisms involved during mechanosensitive cell cycle entry and phenotypic switching. Elucidation of mechanisms by which Notch exploits these processes is of critical importance for understanding both normal VSMC development as well as the underlying causes of significant human vascular conditions such as ATAA. So far, the exact mechanism and more specifically the connection and enhancement/inhibition of pathways involved in mechanosensing and response underlying vascular pathology and the interaction of the ECM with vascular cells in the context of ATAA remains elusive.

## Identified genes in familial ATAA

In recent years, major progress has been made in unraveling gene mutations as molecular markers for predisposition to ATAA. This has been by the identification of a variety of single nucleotide polymorphisms (SNPs) from genome-wide association studies (GWAS) suggestive of having a role in ATAA pathophysiology [165]. Also, genetic mutations in ATAA pathophysiology including systemic features are frequently classified by clinicians as a syndromic disease with clear connective tissue anomalies [129]. In the absence of these features, gene mutations in ATAA are often described as causative of a monogenetic connective tissue disorder, affecting proteins encoding for VSMC contractile apparatus or ECM of the aortic wall (Table 2) [213].

**Table 2:** A Comprehensive overview of key genes in hereditary thoracic aortic aneurysm and dissection

Clinical grade	HTAAD genes panel	Protein	%-pathogenic variant^a^	Syndrome
CAT A1/A2 definitive/ strong	*ACTA2*	Smooth muscle alpha 2 actin	+ + +	Multiple SMC dysfunction syndrome
	*COL3A1*	Collagen alpha 1(III) chain	+ + +	Ehlers-Danlos syndrome type IV
	*FBN1*	Fibrillin-1	+ + +	Marfan syndrome
	*SMAD3*	SMAD family member 3	+ + +	Loeys-Dietz, aneurysms osteoarthritis syndrome
	*TGFβ2*	Transforming growth factor beta-2	+ + +	Loeys-Dietz syndrome
	*TGFBR1*	Transforming growth factor beta receptor 1	+ + +	Loeys-Dietz syndrome
	*MYH11*	Smooth muscle-myosin heavy chain 11	+ + +	Familial aortic aneurysm
	*MYLK*	Myosin light chain kinase	+ + +	Familial aortic aneurysm
	*LOX*	Lysyl oxidase	+ +	Not yet classified
	*PRKG1*	Protein kinase, cGMP-dependent 1	+ +	Familial aortic aneurysm
	*EFEMP2*	Fibulin 4	+ +	Cutis laxa type Ib
CAT B moderate/ limited	*ELN*	Tropoelastin	+	Cutis laxa, Williams Syndrome
	*FBN2*	Fibrillin-2	+ + +	Congenital contractural archnodactyly
	*FLNA*	Filamin A	+ +	Periventricular nodular heterotopia
	*NOTCH1*	Notch1	+ +	BAV with aneurysm
	*SLC2A10*	Glucose transporter protein type 10	+ + +	Arterial tortuosity syndrome
	*SMAD4*	SMAD family member 4	+ +	JP/HHT syndrome
	*SKI*	SKI proto-oncogene	+ + +	Shprintzen-Goldberg syndrome
	*TGFB3*	Transforming growth factor beta-3	+ +	Loeys-Dietz syndrome
Undetermined	*BGN*	Small leucine-rich proteoglycan	+ +	Meester-loeys syndrome
	*FOXE3*	Forkhead box E3	+ +	Familial aortic aneurysm
	*MAT2A*	Methionine adenosyltransferase IIA	+ +	Familial aortic aneurysm
	*MFAP2*	Microfibril-associated protein 2	+ +	Familial aortic aneurysm
	*SMAD2*	SMAD family member 2	+	Not yet classified

^*a*^*%- pathogenic variant: (* +*) 1–25%; (*+ +*) 26–75%; (*+ + +*) 75–100%*

### Genome-wide association studies identifying SNPs in ATAA

In the context of GWAS, various SNPs have been discovered to be associated with either a decreased or elevated risk of ATAA progression. In addition, several novel genes, including *CD40* [36], *ESR1* [233], *COQB1* [109], *ENTPD1, PDLIM5* (PDZ and LIM domain 5), *ACTN4* (alpha-actinin-4)*, and GLRX* [33] have been identified in the context of ATAA.

The association of ESR1 with CD8 + T-cells has been identified as a positive correlate with ATAA [233]. However, exploring the reduced inflammatory pathogenesis in ATAA requires additional investigation. The mechanistic involvement of COQB1 in the association with ATAA is explained by the negative feedback of rs386542, which elevates COQB1 expression. This increase in COQB1 expression leads to heightened VSMC metabolic activity, ultimately resulting in a decreased risk of ATAA [109].

The increased risk of developing ATAA has been linked to genetic loci, with several genes implicated in predisposing to ATAA, including *VKORC1, CTNNA3, FRMD6, MBP, TCF7L2, TGF-B2,* and *FBN1* [8, 82, 113, 178, 189, 212]. Further, the genetic loci of 9q21, 18q11, 15q21, and 2q35, have been identified as risk regions in ATAA. Interestingly *FBN1*, the predominate genetic source (including gene loci 15q21) of Marfan syndrome has been identified by multiple studies on separate populations as strongly associated SNPs with ATAA progression and development [82, 113, 212].

GWAS has pinpointed various shared factors in the development of ATAA, whether involving VSMC or ECM roles. However, apart from FBN1, there has been limited subsequent exploration to reinforce earlier discoveries of the ATAA association. Notably, no GWAS has identified markers related to VSMC contractility as implicated in ATAA.

### Mutations in genes encoding for the contractile apparatus of VSMCs

VSMCs consist of thin filaments such as α-actin (encoded by *ACTA2*) and thick filament myosin heavy chains (encoded by *MYH11*), connected by two essential light chains (LC) and regulatory light chains. Contraction of VSMCs is initiated by calcium-calmodulin complex (Ca-CaM) and the force is generated by ATP-dependent cyclic interactions between isoforms of α-actin and myosin heavy chain [143]. The impact of *ACTA2* mutations on α-actin function was studied using in vitro assays [124]. Mutant α-actin showed functional defects, such as disrupted force generation and defective contractile VSMC function [68, 124, 146]. Besides *ACTA2* mutations, also *MYH11* mutations have been shown to disrupt cyclic interaction, which is predisposed in ATAA [172, 235]. Interestingly, increased expression of MYH11 increases the risk of ATAAD by approximately tenfold [105]. Mutations in the genes *ACTA2* and *MYH11* are the two most common mutations causing familial ATAAD. However, heterozygous loss-of-function mutations in myosin light chain kinase (*MYLK*) [217] and type I cGMP-dependent protein kinase (*PRKG1*) [69] have been reported in heritable ATAAD as well. Loss-of-function mutations in *MYLK* and *PRKG1* disrupt kinase binding to calmodulin (CaM) and reduce kinase activity. Taken together, this demonstrates that proper VSMC-contractile function is critical for maintaining the integrity of the thoracic aorta throughout life.

### Mutations in genes encoding for ECM proteins

As mentioned above, the contractile apparatus of VSMCs binds microfibrils surrounding elastin fibers through focal adhesions on the cell surface of VSMCs. Fibrillin-1 (encoded *FBN1*), a large glycoprotein, is the major protein in microfibrils. Heterozygous *FBN1* mutations in the gene coding for this protein predispose to ATAAD in patients with Marfan syndrome [113, 182]. Moreover, the use of pluripotent stem cells to model various embryonic origins of VSMCs has revealed an inherent upregulation of *FBN1* expression in neural crest VSMCs that drives the incidence of ATAA in Marfan syndrome [65].

The role of *FBN1* mutations in ATAA has been thoroughly investigated and confirmed that mutations in *FBN1* disrupt the structure and deposition of ECM microfibrils [76, 141]. It has been suspected that the aortic wall of Marfan patients contains low levels of fibrillin-1, which corresponds with findings in undifferentiated VSMCs. [66] These findings have been confirmed in animal studies, where VSMCs from Marfan mice showed VSMC detachment from the ECM causing VSMC phenotypic switching [26] and impaired cytoskeleton and focal adhesion organization [213]. This is supportive of findings from the use of iPSC-VSMCs derived from various lineages, wherein increased expression of *FBN1* in neural crest VSMCs was found. In addition, it was reported that neural crest VSMCs of Marfan harboring mutations to *FBN1* resulted in increased apoptosis compared to VSMC that had been *FBN1* CRISPR corrected as well as to wild-type neural crest VSMCs [65]. It is well known that these signaling pathways are involved in the proliferation, apoptosis, inflammation, and phenotypic switching of VSMCs. Besides *FBN1*, there are other genes known to affect the ECM with pathogenic outcomes, such as *COL5A1/2, COL1A1*, and *COL1A2* in Ehlers–Danlos syndrome [38]. Furthermore, lysyl oxidase (*LOX*) mutations encoding for an enzyme aiding the cross-linking of collagen and elastin in the aortic wall may lead to ATAAD [112].

## The role of aging

Aging is the biggest risk factor for impaired cardiovascular health, with cardiovascular disease being the cause of death in 40% of individuals over 65 years old. The remodeling of the human thoracic aorta correlates with aging [115, 140] and ATAA related to aging is often labeled as a degenerative disease. As a main component of the vessel wall, elastin fulfills a key role in the remodeling process. Elastin content progressively decreases with a half-life of some 75 years in humans [203]. Several studies reported that with progressing age, alterations in the quantity and quality of elastin and collagen cause a decrease in total arterial compliance [208, 225]. Aging is associated with the destruction of interlaminar fibrillar elastic structures as well as a decreased amount of medial VSMCs [208]. This results in a reduction or loss of elastic and vasoactive function of the vascular system [58]. Loss of aortic elasticity is not only related to damage of elastin but also changes in collagen content. As individuals age, collagen type I remains consistently predominant, while the quantity of collagen type III declines gradually from the heart to the distal portion of the aorta [133].

Senescence-associated β-galactosidase (SA-β-Gal) activity is used as a tool for in vivo assessment of aging. The increase of SA-β-Gal is a result of increased lysosomal content, the expression of cyclin-dependent kinase inhibitors, the presence of DNA damage, or the presence of critically short telomere length [44, 63]. Telomere length provides a potential marker for an individual’s biologic age. Several studies suggested that telomerase plays a protective role in AAA [47] and ATAA [7]. Telomeres shortening, reduced telomerase function, and cellular senescence of VSMCs play a crucial role in the development of ATAAs. Significantly higher expressions of stress-induced senescence markers p16(INK4a) and p19(ARF) in telomerase-deficient mice were shown compared to wild-type mice [18]. Further, telomere shortening in human blood leukocytes reveals its use as a potential biomarker for ATAA [12].

Other important macromolecules contributing to the pathogenesis of ATAAD, are glycosaminoglycans (GAGs) and proteoglycans (PG), fundamental contributors to the structure and function of the aortic wall. There is contradictory data regarding changes in GAGs upon aging, most studies reported an increase in GAGs, often followed by a decrease upon further in an aging aorta [16, 154]. In ATAAD, multiple structural disruptions are reported as a result of localized GAG accumulation leading to increased interlamellar pressure [175]. Mitochondria also play an important role in aging. Tyrrell et al. demonstrated that with aging mitochondrial dysfunction may activate innate immune pathways including the TLR9, inflammasome, and STING pathways [210]. Recently, it was demonstrated in mice that the mitochondrial function of VSMCs is controlled by the ECM and drives the development of aortic aneurysms in Marfan syndrome [157].

Alteration in connective fibers within the aorta impairs the elastic recoil and reduces adhesive strength between the aortic wall layers. This may impair the functionality of aortic cells and subsequently lead to ATAA formation or aortic rupture in case pressure-induced wall stresses exceed this strength.

## Sex differences

Sex differences play a significant role in the development, management, and clinical outcomes of aorta pathology. Biologically, women are protected against ATAA due to premenopausal levels of estrogen and therefore often present with ATAA at an older age than men [150]. Although ATAA is less prevalent in women, a recent epidemiologic study demonstrates that women have a 40% increased risk of mortality [152] and a threefold increased risk of ATAAD or rupture compared to men [43]. Although heritable ATAA growth rates were similar, ATAA growth rates were over three-fold higher in women than in men with degenerative ATAA [32]. These differences in sex etiology can be explained by anatomic differences such as aorta size and proportional dilation between genders. Despite the correction of aneurysm size to body size, acute aortic syndromes occur at smaller aneurysm sizes in women than in men with worse ATAA-related outcomes [55]. Thus, a smaller diameter of the aorta can progress more rapidly in women requiring close monitoring. In vitro and animal studies have indicated that estrogen can reduce collagen deposition and increase elastin in the aortic wall, potentially contributing to the prevention of TAA development [150, 168]. However, during and after menopause women exhibit a greater aortic stiffening and impairment of elastic properties, which correlates with declining levels of estrogen [215]. This may explain why women have a more progressed state of aortic disease and need to undergo surgery for ATAA at an older age [15].

## Sporadic and genetic biomarkers in ATAA(D)

### Sporadic or nonfamilial biomarkers

#### Matrix metalloproteinases

MMPs have emerged as valuable circulating markers for ATAA pathology. Studies have highlighted the significance of circulating MMP-1 and MMP-2 [162], MMP-3 [200], and MMP-9 [117] as potential indicators in assessing and monitoring ATAA. Specifically, circulating MMP-3 and insulin-like growth factor binding protein 2 (IGFBP-2) have been associated with aortic diameter in patients with ATAA [200]. Following the acute phase of aortic dissection, there is an increase in circulating MMP-9 levels, with plasma MMP-9 expression reaching its maximum approximately 2 weeks after the onset of symptoms [205]. In addition, increased expression of MMP-1, TIMP-1, and MMP-12 was positively associated with systolic WSS and TAWSS observed in the proximal ascending aorta (Table 3) [162], further underlining the importance of MMPs in assessing and monitoring aortic diseases. In the context of AAA, it is noteworthy that the administration of a pan-MMP inhibitor resulted in a slight exacerbation of aneurysm severity, in terms of aneurysm growth [136]. This suggests that the mechanism underlying MMPs and aneurysm formation/progression is complex and that a targeted approach may be required to effectively modulate MMPs in the context of AAA.

**Table 3:** Promising clinical biomarkers for predicting and monitoring the progression of ascending thoracic aortic aneurysm and dissection

Category	Biomarker	Disease	Source	Number of patients (*n*)	Relation between biomarkers and TAA/D	Author, year
Matrix metalloproteinases	MMP-1	TAA	Plasma	125	Upregulated, (*P* = 0.031)	Pasta S et al. [162]
	MMP-2	TAA	Plasma	125	Upregulated, (*P* = 0.020)	Pasta S et al.[162]; Sangiorgi G et al., 2006
	MMP-3	TAA	Plasma	158	Upregulated, (*P* = 0.019)	Thijssen CGE et al. [200]
	MMP-9	TAA	Serum	79	Upregulated, (*P* < 0.05)	Li T et al., 2018; Sangiorgi G et al., 2006; Maguire et al., 2019
	TIMP-1	TAA	Plasma	125	Upregulated	Pasta S et al. [162]
Serine proteinase inhibitor	A1AT	TAA/ TAAD	Serum	51	Downregulated (*P* = 0.0016)	Dako F et al. [39]
ECM degradation	ACAN	TAAD	Plasma	33	Upregulated, (*p* < 0.001)	König KC et al., [104]
	PG	TAAD	Serum	24	Upregulated	Rai P et al. [170]
	GAG	TAAD	Serum	24	Upregulated	
Microcalcification	AHSG	TAA/D	Serum	14	downregulated, (*p* = 0.0002)	Kazamia R et al. [99]
	miR-574-5p	TAA/D	Serum	28	Upregulated, (*p* < 0.001)	Boileau A et al., [22]
Inflammation	IL-6	TAAD	Plasma	158	Upregulated, (*p* = 0.018)	Meccanici F et al. [135]
	GDF-15	TAAD	Plasma	158	Upregulated, (*p* = 0.006)	Meccanici F et al. [135]
	TLT-2	TAAD	Plasma	158	Upregulated (*P* = 0.00042)	Thijssen CGE et al. [200]
	C18-ceramide	TAAD	Plasma	70	upregulated (p < 0.001)	Yang H et al. [222]
	IL-8	TAA	Serum	52	Upregulated (*p* < 0.0001)	Daskalopoulou A et al. [41]
	ICAM1	TAA	Serum	52	Upregulated (*p* < 0.0001)	Daskalopoulou A et al. [41]
	CCL5	TAA	Serum	52	Upregulated (*p* < 0.0001)	Daskalopoulou A et al. [41]
	HBD1	TAA	Serum	52	Upregulated (*p* < 0.0001)	Daskalopoulou A et al. [41]

*MMP* matrix metalloproteinases, *TIMP* Tissue inhibitors of metalloproteinases, *A1AT* Alpha1 Antitrypsin, *ACAN* Aggrecan, *PG* Proteoglycan, *GAG* Glycosaminoglycans, *AHSG* Alpha-2-HS-Glycoprotein; *IL* = interleukin, *GDF-15* Growth differentiation factor 15, *TLT-2* Triggering receptor expressed on myeloid cell-like transcript 2, *ICAM1* Intercellular adhesion molecule-1, *CCL5* C–C Motif Chemokine ligand 5, *HBD1* Human beta-defensin 1

#### α-1-Antitrypsin protein

α1-Antitrypsin (A1AT) is a circulating serine proteinase inhibitor crucial for maintaining connective tissue integrity. A deficiency in A1AT is characterized by decreased levels, potentially leading to arterial wall degradation due to insufficient protection against the proteolytic effects of elastase and collagenase. Notably, heightened levels of MMP-9 have been identified in the vessel walls of aortic aneurysms, and these levels correlate with aortic diameter [126]. Researchers indicate that A1AT may inhibit MMP-9 activity by deactivating elastase and restraining gelatinase B within neutrophils [90]. The first controlled study investigating the relationship between A1AT deficiency and ascending aortic diameter has recently been published [39]. In this study, serum A1AT levels in the aneurysmal group were approximately 9.5 times lower than those in the nonaneurysmal group [39]. The link between reduced A1AT levels and aortic aneurysm provides additional support for its significance in evaluating the risk of ATAAD (Table 3).

#### Proteoglycans

Plasma levels of aggrecan (ACAN), a multimodular proteoglycan (PG) protein, were significantly enhanced in plasma samples of ATAAD patients compared to samples from healthy patients [104]. Also, increased levels of PG) and glycosaminoglycan (GAG) were detected in the serum of ATAAD patients (Table 3) [170].

#### Desmosine (DES)and isodesmosine (IDES)

As the aorta contains elastin, novel biomarkers for thoracic aortopathies are potentially the breakdown products of elastin: desmosine (DES) and isodesmosine (IDES), which are released in plasma, urine, or sputum [125]. Desmosine plays a pivotal role in cross-linking tropoelastin, offering valuable insights into disease mechanisms [54]. Researchers have investigated the use of desmosine as a biomarker to assess the extent of elastin degradation in the aorta, helping in the diagnosis and monitoring of aortic aneurysm progression [52, 145]. Elevated levels of DES in blood or urine samples may indicate increased elastin turnover, suggesting ongoing damage to the aortic wall. DES and IDES have been previously associated with AAA size, risk of rupture [50, 145, 211], and as a prognostic marker in acute myocardial infarction [5].

Interestingly, when combined with MRI, DES may enable the direct visualization of biologic processes at precise anatomic sites. This has been demonstrated in a Marfan mouse model [155]. Currently, there is a lack of available data on DES/IDES in ATAA. The exploration of plasma DES concentrations in studies to predict dissection or rupture in thoracic aortopathy holds significant value (Table 3).

#### Microcalcification

An association has been identified between reduced levels of alpha-2-HS-glycoprotein (AHSG), also known as Fetuin-A, in human blood plasma, as determined through mass spectrometry-based proteomic analysis, and an increased risk of ATAA formation [99]. AHSG binds to calciprotein particles (CPPs), forming essential complexes for regulating mineral metabolism. This interaction is essential for stabilizing and facilitating the clearance of calcium and phosphate from the circulation [106].

Plasma AHSG concentrations can differentiate between patients with ATAA and healthy controls [99]. AHSG deficiency is associated with inflammation and links vascular calcification to mortality in patients on dialysis [101]. This suggests that it might be a promising bloodborne biomarker for early ATAA diagnosis. It is worth noting that during the vascular calcification process, VSMCs may undergo phenotypic changes from a synthetic state to a chondrogenic state, which may be accompanied by the release of EVs in the bloodstream [96]. Small extracellular vesicle-derived miR-574-5p was significantly up-regulated in the serum of patients with ATAA compared to the control, and this up-regulation was higher in patients with large aneurysms (> 49 mm) [22].

Furthermore, circulating dp-ucMGP has been associated with elastin degradation, although it has not been studied in the context of ATAA [164, 188]. Findings indicate that dp-ucMGP serves as a potential biomarker for identifying individuals at risk of developing arterial and valvular calcification, suggesting its potential utility in the clinical assessment of diseases before their clinical manifestation. Although the association between dp-ucMGP and ATAAD remains unclear, it's worth noting that vitamin K deficiency, as indicated by dp-ucMGP, correlates with circulating plasma DES and IDES levels in both CVD and COPD [166]. This unexplored avenue presents an opportunity for further research to elucidate the potential implications of circulating dp-ucMGP in the context of ATAA, bridging the gap between vitamin K deficiency, vitamin K antagonist use, and elastin degradation. In addition, the conjugation of circulating dp-ucMGP with 18F-NaF PET presents the potential to develop a non-invasive imaging tool capable of precisely quantifying and colocalizing active micro-calcification within the arterial wall. This innovative approach holds promise for advancing our understanding of micro-calcification dynamics and its role in vascular health (Table 3).

#### Inflammation

Although the role of inflammation in ATAA is currently insufficient, several markers suggest activation of the innate immune system and the subsequent development of a low-grade chronic inflammatory reaction, which may lead to the evolution of ATAA. In this cross-sectional study, the blood of 158 patients with ATAAD was analyzed, and several circulating blood biomarkers were associated with the maximal thoracic aortic diameter estimated by CT-angiography or transthoracic echocardiography. The biomarkers that were found to be significantly associated with aortic size were primarily inflammatory markers IL-6 and GDF-15 [135]. Another study associated elevated levels of TLT-2 expressed in cells of the immune system [200] and IL-11[226] with ATAAD. Yang et al., demonstrated an increase in C18-ceramide in ATAAD, suggesting its role in aortic inflammation via association with NLRP3 in the NLR family [228]. Moreover, a novel comparison of a targeted proteomic approach has shown that patients with ATAA have increased serum levels of several inflammatory markers, such as IL-8, intracellular adhesion molecule-1 (ICAM1), C–C motif chemokine ligand 5 (CCL5), and human beta-defensin 1 (HBD1) (Table 3) [41].

### Genetic biomarkers

Over the last 2 decades, there has been an emergence of newly discovered causative genes and syndromes associated with subtle or even non-existent external phenotypes. Genetic heterogeneity of hereditary ATAAD has been established by the ClinGen Aortopathy Working Group [173]. The genes were selected based on the published data and genes tested on clinical aortopathy gene panels that are currently available. Out of the 53 genes subjected to testing, the following 11 genes were conclusively identified as having a definite association with heritable ATAAD, and are clinically actionable listed in highly penetrant risk category (A1): *ACTA2, COL3A1, FBN1, MYH11, SMAD3, TGF-B2, TGFBR1, TGTBR2, MYLK, LOX, PRKG1* [173]. These genes were identified over three years ago, and their association with ATAAD has been well-documented [25, 70, 142]. These genes play a role in encoding proteins associated with contraction and adhesion of VSMCs to ECM. In additiony, they contribute to TGF-β signaling pathways and VSMC metabolism. Recently, novel genes have been discovered. Tomida et al. [201] unveiled a previously overlooked mechanism connecting familial thoracic aortic aneurysm and dissection to impaired calcium ion uptake by *MYH11*, suggesting that elevating cytosolic Ca2 + levels could potentially prevent ATAAD [201]. Two additional studies have reported evidence suggesting that specific genetic variation at the rs2118181 locus within the *FBN1* gene may be associated with an increased risk of developing ATAAD (Table 3) [82, 113].

## Future outlook

Significant progress has been made over the last decades in understanding the pathophysiology of ATAA. However, important gaps remain in the early detection of acute aortic pathologies such as ATAAD. The autopsy reports indicate that up to 25% of patients with ATAAD die before diagnosis [158], and these cases often involve younger individuals [167]. Relying solely on aortic diameter for risk assessment is inadequate to distinguish between different pathologic processes with varying risks of acute complications.

Most of the recent literature on ATAA focused on identifying circulating biomarkers to improve diagnosis. While these biomarkers show promising results, their isolated use may lack specificity in indicating disease progression due to their involvement in multiple processes throughout the body. Understanding the patient-specific cellular and molecular mechanisms and integrating complementary diagnostic tools by combining circulating biomarkers, with advanced imaging tools, such as molecular imaging probes could enable direct visualization of biologic processes at specific anatomic locations.

Further investigation emphasizes the need for more personalized strategies to improve risk assessment such as integrating imaging data with genotypes and circulating biomarkers to identify patients at high-risk and guide surgical decision-making.

## Abbreviations

AAA: Abdominal aortic aneurysm
ACTA2: Actin Alpha 2, Smooth Muscle
ACA: Aggrecan
ADmax: Absolute max diameter
AHA: American Heart Association
AHSG: Alpha-2-HS-glycoprotein
A1AT: α1-Antitrypsin
ATAA: Ascending Thoracic Aortic Aneurysm
ATAAD: Acute type A aortic dissection
BAV: Bicuspid aortic valve
CADESIL: Cerebral autosomal dominant arteriopathy with subcortical infarcts and leukoencephalopathy
COL3A1: Collagen type III
dp-ucMGP: Dephosphorylated undercarboxylated Matrix Gla protein
DES: Desmosine
ECM: Extracellular matrix
ECs: Endothelial cells
ELN/COL: Elastin-to-collagen
FBN1: Fibrillin 1
18F-FDG: 18F-fluorodeoxyglucose
18F-NaF: 18F–sodium fluoride
GAGs: Glycosaminoglycans
hiPSCs: Human-induced pluripotent stem cells
IDES: Isodesmosine
KLF4: Krüppel like factor 4
LDS: Loeys–Dietz Syndrome
LVOT-angle: Left ventricular outflow tract angle
MMP: Matrix metalloproteinases
MYH11: Myosin heavy chain 11
MYLK: Myosin light chain kinases
MGP: Matrix Gla-protein
NICD: Notch intracellular domain
PECAM-1: Platelet EC adhesion molecule
PG: Proteoglycans
PRKG1: Protein kinase cGMP-dependent type 1
RANTES: Regulated on activation, normal T-cell expression, and secreted
ROS: Reactive oxygen species
SA-β-Gal: Senescence-associated β-galactosidase
TGF-β: Transforming growth factor-β
TAV: Tricuspid aortic valve
VE-cadherin: Vascular endothelial-cadherin
VEGFR2: Vascular endothelial growth factor receptor 2
VKOR: Vitamin K epoxide reductase
VSMC: Vascular smooth muscle cell
vWF: Willebrand factor
WSR: Wall shear rate
WSS: Wall shear stress
